# MicroRNA-133b inhibits the migration and invasion of non small cell lung cancer cells via targeting FSCN1

**DOI:** 10.3892/ol.2021.12929

**Published:** 2021-07-15

**Authors:** Xucheng Yang, Pengfei Lei, Yong Huang, Zijian Zhang, Yingying Zhang

Oncol Lett 12: 3619-3625, 2016; DOI: 10.3892/ol.2016.5044

Following the publication of this paper, it was drawn to the Editors’ attention by a concerned reader that the scratch-wound assay data shown in Figs. 4B and 5B were strikingly similar to data appearing in different form in other articles by different authors. Owing to the fact that the contentious data in the above article had already been published elsewhere, or were already under consideration for publication, prior to its submission to *Oncology Letters*, the Editor has decided that this paper should be retracted from the Journal. The authors were asked for an explanation to account for these concerns, but the Editorial Office did not receive any reply. The Editor apologizes to the readership for any inconvenience caused.

